# Supplementary material for: Patterns of Intron Gain and Loss in Fungi
Source: PLoS Biol. 2004 Nov 30;2(12):e422. doi: 10.1371/journal.pbio.0020422 (PMC532390; doi:10.1371/journal.pbio.0020422)
Supplement: Table S1 — Also available at http://genes.mit.edu/NielsenEtAl/. (4.3 MB ZIP). [file pbio.0020422.st001.zip › NielsenEtAl/html/1400.html]

AN0111.1.NCU08036.1.MG07087.1.FG04284.1


```
 CLUSTAL W (1.82) Multiple Sequence Alignments - Introns Inserted


Sequence 1: NCU08036.1	829 aa
Sequence 2: MG07087.1	816 aa
Sequence 3: FG04284.1	823 aa
Sequence 4: AN0111.1	851 aa
Alignment Length: 857 aa
Number Identitical Residues: 562 aa
Alignment Score (without introns) 25083


MG07087.1 	----------------MHLV0TDDDFVYEQDIQRSPGSTKPWLAYISYKIQHGTVEEQAF
NCU08036.1	---MSILSRVSDRRPDLSLV0SEEDFPYEQDIVRNPGSTKPWLAYIEYKLQKGTVQEQAY
FG04284.1 	MAPIVPALSWACRQPQLHLV0SNEDSVYEQDILRDPGSVKPWLTYIEFKTRHGSILEQAY
AN0111.1  	------MDRSSASRPDLYLI0ADNDSVYEQDLLRNPGTIKPWLAYIEYKQQNGTLYEQAF
          	          :   ..: *: :::*  ****: *.**: ****:**.:* ::*:: ***:

MG07087.1 	V-----------------LERACMQLPRSYKLWKM0YLTFRTKHIAKLNAAIFAAEYRKV
NCU08036.1	I-----------------MERACVQLPRSYKLWKM0YLRFRTKHVSKLNAAIFASEYQKV
FG04284.1 	V-----------------MERACTQLPRSYKLWKL0YLSFRVKHVTKLNSALFDKEYRKV
AN0111.1  	VGRPLDALIISILNDFQVMERACKQLPRSYKLWKM0YLEFRTKHLKNRNAIKYRAEFQKV
          	:. . .:   :  .. . :**** **********: ** **.**: : *:  :  *::**

MG07087.1 	NSLFERALILLNKMPRIWEMYLKFLMKQPLVTLTRRTFDRALRALPITQHNRIWALYRPF
NCU08036.1	NSLFERALILLNKMPRIWEMYLKFLMQQPLVTHTRRTFDRALRALPITQHNRIWALYRPF
FG04284.1 	NALFEKALNLLHKMPRIWEMYLRFLTKQPLVTLTRHVFDRALRALPITQHNRIWAIYRPF
AN0111.1  	NTLFERALILLNKMPRIWEMYLTFMLQQPLVTQTRRTFDRALRALPVTQHNRIWKLYKTF
          	*:***:** **:********** *: :***** **:.*********:******* :*:.*

MG07087.1 	ANSAAGPTAVKIWRRYMQVHPEDAEDFIELLYQVGYYTEAAKKYIDVLNNPRFTSKHGKG
NCU08036.1	ANSAEGETAVKIWRRYMQVHPEDAEDFIELLVAVGLYTEAVHKYIEILNNPRFTSKNSKG
FG04284.1 	ANSAAGVTAVKIWRRYMQVHPEDAEDFIELLVQNGLYTEAALKFIEVLNNTRFNSKHGKG
AN0111.1  	ARSASGQTAVKIWARYMQIHPENAEEYINLLVEMGHYTDAIKRYMEILDNPRFQSREGKS
          	*.** * ****** ****:***:**::*:**   * **:*  :::::*:*.** *:..*.

MG07087.1 	HFELWSEMVELLVEHATEVEAGYETGIDVERIIRSGIERFADQRGKLWVGLATYWIRRGS
NCU08036.1	HYELWSEMVDLLVEHATAVETGHETGIDVERIIRSGIERFADQRGKLWCGLATYWIRRGS
FG04284.1 	HYELWSEMVDLLVAHAADIETGHETGIDAERIIRSGISRFADQRGKLWSGLATYWIRRGS
AN0111.1  	NFQLWTEMVDLLVSKAKKIETGPQTGIDVDAILRSGIDRFADQRGKLWAGLATYWITKGN
          	:::**:***:*** :*  :*:* :****.: *:****.********** ******* :*.

MG07087.1 	FERARDVFEEGITTVMTVRDFTLVFDSYAEFEESVIGAMMEVAGQRAEKGVVDEAADFDL
NCU08036.1	FERARDVFEEGITTVMTVRDFTLVFDSYTEFEESIISALMEMASTRAEKGEVDEVADFDL
FG04284.1 	FERARDVFEEGITTVMTVRDFTLIFEAYTEFEESIIGALMEVASGRAEKGVEDENADFEL
AN0111.1  	FEKARDVFEEGITTVMTVRDFTLIFDSYVEFEESIIGSLMEAAAVRADNGKADEEADFDL
          	**:********************:*::*.*****:*.::** *. **::*  ** ***:*

MG07087.1 	DIRMMRFEHLMDRRPFLLNDVLLRQNPNNVNEWEKRVALWGDNHNEVVNTYTNAIAAVQP
NCU08036.1	DIRMMRFEHLMDRRPFLLNDVLLRQNPNNVTEWEKRVALWGDNKEEVVKTYLDAIEAIQP
FG04284.1 	DIRMMRFEQLMDRRPFLLNDVLLRQNPNNVPEWEKRVALWGDNKKEVAQTYTDAIAAIQP
AN0111.1  	DLRMLRFEQLMDRRPFLVNDVLLRQNPNNVIEWEKRVALWGDNNVEIVNTYTAAIAAINP
          	*:**:***:********:************ ************: *:.:**  ** *::*

MG07087.1 	KKASGPFHQLWANYAKFYERGGDLRSARIIMEKAVKVPFKSVVELADMWIEWAEMELRND
NCU08036.1	KKAVGALHQLWTNYAKFYEAGGDLSSARRIMEKAVKVPYKSVAELADMWIEWAEMELRNE
FG04284.1 	KRAVGAFHQLWTNYAKFYERGGDLRNARVIMEKAIKVPFKSVAELADMWIEWAEMELRNE
AN0111.1  	KKAVGKFSELWVNYAKFYERGGDLDTARIIFEKAVKVPFKSVNELAETWCEWAEMELRSE
          	*:* * : :**.******* **** .** *:***:***:*** ***: * ********.:

MG07087.1 	NFEEAVRIMAKAVQAPKRSTVDYFDETLTPQQRVHKSWKLWSFYVDLVESVGTLEDTKKV
NCU08036.1	CFDEAMKVMAKAVQAPKRSTVDYFDETLSPQQRVHKSWKLWSFYVDLVESVSSLDETRKV
FG04284.1 	NFDDAVRIMAKAVQAPKRSTVDYFDETLSPQQRVHKSWKLWSFYVDLVESVSTLDETKKV
AN0111.1  	NFDKAVEIMAKATQAPKKSTVDYFDETLSPQQRIHKSWKLWSFYVDLVESVSSIEETKKV
          	 *:.*:.:****.****:**********:****:*****************.::::*:**

MG07087.1 	YERIFELRIATPQTVVNYANLLEEHKYYEESFKIYERGLDLFSYPVAFELWNMYLTKAVD
NCU08036.1	YERIFELRIATPQTVVNYANLLEEHKYFEESFKIYERGLDLFSYPVAFELWNLYLTKAVD
FG04284.1 	YERIFELRIATPQTVVNYANLLEEHKYYEESFKIYERGLDLFSYPVAFELWNLYLTKAAD
AN0111.1  	YERIFELRIATPQTVVNYANLLEEHKYFEESFKVYERGLDLFTYPVAFELWNLYLTKAVD
          	***************************:*****:********:*********:*****.*

MG07087.1 	RKISIERLRDLFEQAVEGCPPKFAKIIYLMYGNLEEERGLARHAMRIYERATRAVSDEDR
NCU08036.1	RKISIERLRDLFEQAVEDCPPKFAKVIYLMYGNLEEERGLARHAMRIYERATRAVADEDR
FG04284.1 	RKIGIERLRDLFEQAVEDCPPKFAKTIYLMYGNLEEERGLARHAMRIYERATRAVADEDR
AN0111.1  	RKIGIERLRDLFEQALDGCPPKFARPLYLMYGNLEEERGLARHAMRIYERATRAVSDEDR
          	***.***********::.******: :****************************:****

MG07087.1 	ADMFNFYITKSASNFGLPSTRQIYERAIAALPDDEARDMCLKFADMEKRLGEIDRARAIY
NCU08036.1	ADMFNFYITKSASNFGLPSTRPIYERAIAALPDAEARDMCLKFADMEKRLGEIDRARAIY
FG04284.1 	ADMFNFYITKSASNFGLTSTRPIYERAIAALPDVEAKDMCLKFADMEKRLGEIDRARAIY
AN0111.1  	FEMFEFYITKSASNFGLPSTRPIYERAIAALPDHEAKEMCLKFAEMERRLGEIDRARAIY
          	 :**:************.*** *********** **::******:**:************

MG07087.1 	GHASQFCDPRTTPAFWTKWEQFEVQHGNEDTFKEMLRIKRAVQAKYN~TDVNFIASQALA
NCU08036.1	GHASQFCDPRTNPGFWTKWDQFEVQHGNEDTYKEMLRIKRSVQAQYN~TDVNFIASQALA
FG04284.1 	GHASQFCDPRTSPDFWTKWEQFEVQHGNEDTFKEMLRIKRSVQAQYN~TDVNFIASQALA
AN0111.1  	GHASQFCDPRTNAPFWQKWEAFEVQHGNEDTFKEMLRIKRSVQAQYN2TDVNFIASQAIA
          	***********.. ** **: **********:********:***:** **********:*

MG07087.1 	RSQQQ--KQANGHGSGAEDGDDGVVDAMEALERQARAPAGFVAATEGNIKGDAATTN---
NCU08036.1	RSQQKR-MEEEAAGNGGGEMDAEVADAMAQLERQARAPVGFVAASEG-PKGGSMPVQ---
FG04284.1 	RSQRQ----------PEGAEDAEITDAMAALERQSRAPQGFVAASTG-PVGGGNTAT---
AN0111.1  	RSQQRAPEGEEATAAAEREMDTETTDAMAALERQARAPIGFVAASTGPEGGNRAPPAGQG
          	***:: .   .. .      *   .***  ****:*** *****: *   *.  .  ...

MG07087.1 	-TTVAANPDAIDIDDMED--
NCU08036.1	-PVEVHNPDAIDLDEMDE--
FG04284.1 	-TAVPSNPDAIEIDGIDE--
AN0111.1  	PVAAPANPDAIDLDDDMEAD
          	. .   *****::*   ::.
```
